# Centrally acting anticholinergic drug trihexyphenidyl is highly effective in reducing nightmares associated with post‐traumatic stress disorder

**DOI:** 10.1002/brb3.2147

**Published:** 2021-05-14

**Authors:** Katsumasa Sogo, Masanobu Sogo, Yoshie Okawa

**Affiliations:** ^1^ Sogo PTSD Institute Medical Corporation Sogokai Hiroshima‐city Japan

**Keywords:** anticholinergic agents, neurotransmitters, PTSD, treatment

## Abstract

**Introduction:**

Following a case study on scopolamine butyl bromide, an anticholinergic drug, we studied the effect of a central anticholinergic drug on post‐traumatic stress disorder (PTSD)‐related flashbacks and nightmares.

**Methods:**

We administered trihexyphenidyl (TP) to 34 patients with refractory PTSD‐related nightmares and flashbacks (open‐label trial [*n = *22]; single‐blind trial [*n = *12]), who had previously received psychiatric treatment for approximately 2–15 years, without therapeutic benefits. The effect of TP was determined using the Clinician‐Administered PTSD Scale (CAPS) and the Impact of Event Scale‐Revised (IES‐R).

**Results:**

Overall, most patients reported an improvement to none or mild on the CAPS for nightmares (88%) and flashbacks (79%).

**Conclusion:**

This study is the first to demonstrate the potential efficacy of TP in the treatment of refractory PTSD‐related nightmares and flashbacks. Further double‐blind, randomized control trials are needed to explore the potential clinical benefits of TP in PTSD.

## Graphical abstract

Central anticholinergic agent (trihexyphenidyl) is very effective and fast‐acting against hallmark symptoms: nightmares and flashbacks of PTSD and its mechanism of action.

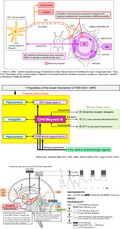

## 1 INTRODUCTION

Post‐traumatic stress disorder (PTSD) is an anxiety disorder that can develop after a traumatic event (e.g., car accidents, violence, and sexual abuse). The fifth edition of the Diagnostic and Statistical Manual of Mental Disorders (DSM‐5™) by the American Psychiatric Association (2013) lists 27 symptoms as diagnostic criteria for PTSD. Among these symptoms, nightmares and flashbacks are considered hallmarks. The DSM‐5 states that in the United States, the projected lifetime risk of PTSD based on the DSM‐5 criteria at 75 years of age is 87%; its 12‐month prevalence for adults in the United States is approximately 3.3%, which is relatively lower in other countries (approximately 0.5%–1.0%). Foa et al. (2009) reported that the lifetime prevalence of PTSD was 1.4% in women and 5% in men. However, it varies across countries and is affected by various cultural and environmental differences (Atwoli et al., 2015).

Nightmares are typically lengthy, story‐like sequences of dream‐based imagery that seem real and incite anxiety, fear, or other dysphoric emotions (American Psychiatric Association, 2013). Nightmares that occur after traumatic experience may replicate threatening situations. They occur almost exclusively during rapid eye movement (REM) sleep (Pagel, 2000), whereas dreams occur during all stages of sleep. Although nightmares are common, recurrent nightmares are the most well‐defined symptoms of PTSD; they differ from night terrors, which are arousal disorders that occur most frequently in children (Van Horn & Street, 2019). Schoenfeld (2012) reported that sleep disturbance is among the most commonly reported symptoms of PTSD. Despite the progress in the development of more specific treatments for sleep disturbance in PTSD, insomnia and nightmares have not been fully resolved. Nielsen (2017) partially described the onset mechanism of nightmares; stress acceleration due to nightmares and alterations in normal emotion regulation (Gross, 2008) lead to increased fear sensitivity and decreased fear extinction.

The mechanisms underlying flashbacks and nightmares have not yet been elucidated, and several classes of drugs, including antidepressants and antipsychotics, are used to treat PTSD. Ishikawa et al. (2019) reported that memantine (an NMDA receptor antagonist) has a neurogenesis‐enhancing effect; it reduces traumatic memories and improves PTSD‐like behavior in mice, including avoidance and anxiety‐like behavior. Ramaswamy (2015) reported that memantine administration to 26 veterans with PTSD and cognitive impairment for 16 weeks resulted in a significant improvement in RBAN (a measure of attention, language, visuospatial skills, and immediate and delayed memories). However, there was no reduction in the recurrence rates. Sasaki et al. (2013) reported that ifenprodil, a neuroprotective agent that binds to the GluN2B subunit of the NMDA receptor, was effective against PTSD‐related flashbacks, and it improved the flashback symptoms by 60% after 3 weeks of administration. Morikawa et al. (2018) suggested that KIF21B, a molecular motor, mediates fear extinction, which provides a new avenue for novel treatments for PTSD.

In 2009, we encountered Patient A, a 21‐year‐old female patient who had suffered severe R‐PTSD‐related flashbacks and nightmares for 9 years. The patient showed no response to long‐term polypharmacotherapy with conventional drugs. Patient A was diagnosed with bacterial diarrhea at another hospital based on abnormal pain and was administered a drip infusion containing antibiotics and scopolamine butyl bromide (SB), which is a peripheral anticholinergic (Wick, 1951). SB had a low blood–brain barrier (BBB) penetration rate (0.01%) in rat experiments (Wahl, 1984; Boehringer Ingelheim Co., Ltd., in‐house document). Remarkably, severe flashbacks resolved approximately 20 min after SB administration. This clinical finding is typically highly unexpected. SB does not readily cross the BBB; however, due to the refractory nature of her symptoms and the ineffectiveness of her previous treatment, the patient requested additional treatment. After explanation and obtaining patient consent, the drug was changed to trihexyphenidyl (TP), a central anticholinergic drug released 66 years ago, which has a greater BBB permeability than SB. Acetylcholine (ACh) is a widely distributed neurotransmitter that regulates the limbic and reticular activating systems involved in sleep and nightmares (Mesulam, 2013). Anticholinergic drugs, however, have not been widely tested for PTSD or specifically examined for treating PTSD nightmares and flashbacks. Based on the experience of Patient A, we explored the potential use of TP as a novel treatment for PTSD‐related nightmares and flashbacks.

## 2 RESULTS

Based on our experiences with Patient A, we enrolled patients with PTSD‐related nightmares and flashbacks in an open‐label and single‐blind study series to evaluate the potential therapeutic effect of TP. No adverse effects other than a floating feeling during the early days of administration (*n = *2) were observed. Most patients were stabilized 2–3 weeks after TP administration, but the symptoms of PTSD (primarily nightmares and flashbacks) worsened upon encountering stress similar to past traumas. Therefore, TP was administered as a single dose after the initial treatment period, when needed.

Because of the small overall sample and exploratory nature of this study, we present the results from all patients in aggregate. A total of 34 participants were treated in a single‐blind (*n =* 12) and open‐label (*n =* 22) study. Medical observations were performed over 9 years before this study. The Clinician‐Administered PTSD Scale (CAPS)‐5 subscores (Weathers et al., 2013) and IES RR were calculated before and after treatment.

### 2.1 Effect of TP treatment on nightmares and flashbacks

Within 2 weeks of treatment, we observed a notable effect on nightmares and flashbacks (Table 1). We found that after TP administration, the patients experienced improvement within 2–3 days. Improvement in CAPS and IES‐R scores was tested using the Wilcoxon signed‐rank‐sum test, which showed a significant impact of TP (Table 2). Across both studies, the mean B2F (nightmare frequency) improved from 3.24 (several times a week, almost daily) before treatment to 0.45 (never, once, or twice a month), and this improvement was significant in both studies (single‐blind *p* = .001; open‐label *p* < .001). Similarly, the mean B2I (nightmare intensity) improved from 3.45 (severe, extreme) before treatment to 0.56 (none or mild) after treatment (single‐blind *p* = .001; open‐label *p* < .001). The mean B3F (flashback frequency) also improved from 3.36 (several times a week, almost daily) before treatment to 0.48 (never, or once or twice a month) after treatment (single‐blind *p* = .001; open‐label *p* < .001). The mean B3I (flashback intensity) improved from 3.32 (severe, extreme) before treatment to 0.61 (none or mild) after treatment across both studies (single‐blind, *p* = .001; open‐label, *p* < .001).

**TABLE 1:** Descriptive summaries of trihexyphenidyl administration to patients between 2012 and 2019 in an open‐label and in a single‐blind trial

Study design	Trihexyphenidyl administration	Variable	Mean	Minimum	Maximum
Single‐blind (n = 12)	Before	B2F	3.58	2	4
		B2I	3.58	2	4
		B3F	3.42	2	4
		B3I	3.5	2	4
		IES‐R	72.42	51	86
	After	B2F	0.58	0	3
		B2I	0.67	0	3
		B3F	0.5	0	2
		B3I	0.67	0	3
		IES‐R	46.25	20	65
Open‐label (n = 22)	Before	B2F	2.91	1	4
		B2I	3.23	2	4
		B3F	3.32	0	4
		B3I	3.14	0	4
		IES‐R	74.95	54	88
	After	B2F	0.32	0	2
		B2I	0.45	0	3
		B3F	0.45	0	2
		B3I	0.55	0	2
		IES‐R	34.36	18	62

Abbreviations: B2F, nightmare frequency; B2I, nightmare intensity; B3F, flashback frequency; B3I, flashback intensity; IES‐R, Impact of Event Scale‐Revised.

**TABLE 2:** Results for the test for the difference in the scores of the various measurements after and before administration of trihexyphenidyl or placebo

Study design	Variable	Mean difference^a^	*p*‐ value^b^
Single‐blind (n = 12)	B2F	−3.00	.001
	B2I	−2.92	.001
	B3F	−2.92	.001
	B3I	−2.83	.001
	IES‐R	−26.17	.0005
	B2F_placebo	−0.25	.250
	B2I_placebo	−0.33	.125
	B3F_placebo	−0.17	.500
	B3I_placebo	−0.23	.250
	IES‐R_placebo	−3.00	.0156
Open‐label (*n =* 22)	B2F	−2.59	<.0001
	B2I	−2.77	<.0001
	B3F	−2.86	<.0001
	B3I	−2.59	<.0001
	IES‐R	−40.59	<.0001

Abbreviations: B2F, nightmare frequency; B2I, nightmare intensity; B3F, flashback frequency; B3I, flashback intensity; IES‐R, Impact of Event Scale‐Revised.

^a^ Mean difference of the mean of the difference in the variables after and before administration of trihexyphenidyl or placebo.

^b^ The *p*‐values are from the Wilcoxon signed‐rank‐sum test using paired observations within a patient.

Patients in both studies reported improvements in IES‐R, from an aggregate average of 73.7 points before treatment to 40.3 Â points after 2 weeks of TP treatment (*p* < .001 for each study separately). The definitions of complete remission and partial remission were determined using the HAM‐D (Frank et al., 1991) and the three‐type scoring system previously described by Andreasen et al. (2005) for depression and schizophrenia, respectively. There is no definition of PTSD remission (Prien et al., 1991). Therefore, efficacy was defined as complete remission of PTSD with a CAPS score of 0 (never, none) or 1 (once or twice a month, mild). Across both studies, the efficacies of TP for nightmares only, flashbacks only, and both nightmares and flashbacks were defined as 0 (never, none) or 1 (once or twice a month) points for B2F and B3F. For nightmares, 23 individuals reported 0 points, 7 individuals reported 1 point, and the overall efficacy rate was 30/34 (88%). For flashbacks, 25 individuals reported 0 points and 2 individuals reported 1 point. The efficacy was higher than expected in 27/34 (79%) patients. For both nightmares and flashbacks, which are hallmarks of PTSD, 19 individuals reported 0 points and 5 individuals reported 1 point. The overall efficacy rate was 24/34 (71%).

Because of the potential impact of study design on the results, we examined the effect of TP treatment and study design on aggregated values using ordered logistic regression. For CAPS scores, there was a significant effect of TP in reducing B2F, B2I, B3F, and B3I, but no significant effect of study design (Table 3). For the IES‐R, linear regression was used to determine the effect of study design and TP. This analysis found a significant interaction between TP treatment and study design (*p* < .02), suggesting that the effect of TP on IES‐R depends on whether the patient was in the open‐label versus. single‐blind study.

**TABLE 3:** Odds ratio estimate for the effect of trihexyphenidyl treatment and design type on B2F, B2I, B3F, and B3I from ordered logistic regression

Measurements	Effect of trihexyphenidyl Odds ratio (95% CI)	Design (open‐label versus. single‐blind) Odds ratio (95% CI)
B2F	0.006 (0.001; 0.031)	0.366 (0.133; 1.005)
B2I	0.004 (< 0.001; 0.025)	0.474 (0.175; 1.286)
B3F	0.007 (0.001; 0.037)	0.938 (0.327; 2.694)
B3I	0.11 (0.003; 0.049)	0.702 (0.254; 1.936)

Abbreviations: B2F, nightmare frequency; B2I, nightmare intensity; B3F, flashback frequency; B3I, flashback intensity.

## 3 DISCUSSION

In a single patient, we observed that the administration of SB, a peripheral anticholinergic drug that does not readily pass the BBB, unrelated to PTSD, rapidly blocked chronic flashbacks and nightmares. This coincidental case led to the hypothesis that a central‐acting anticholinergic agent with high BBB permeability, such as TP, should demonstrate an even stronger effect on flashbacks and nightmares, two hallmark symptoms of PTSD. Among the 34 patients with R‐PTSD, the administration of TP exhibited 71%–88% efficacy in the treatment of flashbacks and nightmares. In addition, the onset of action was rapid, and the effects were observed 1–1.5 hr after administration. This rapid onset of action is a critical and major benefit for patients. Current medications used for PTSD require 2–3 weeks after administration to take effect. In comparison, TP shows effectiveness between the second and third day and 1 week after administration.

Patient A was intravenously administered an antibiotic and SB, but the anticholinergic drug likely had a greater effect on the flashbacks and nightmares. SB does not readily cross the BBB; however, it has a strong clinical effect. The brain of Patient A was likely in an abnormally excited state due to severe flashbacks and nightmares. Brain regions likely to be activated include the amygdala, hippocampus, and cortex, which are connected bidirectionally to the cholinergic basal ganglia, medial septum, diagonal band of Broca, and Meynert nucleus basalis. Abnormal excitation of the Meynert nucleus basalis may stimulate the muscarinic receptors of astrocytes and capillary endothelial cells, leading to the transformation of BBB permeability (Arita, 2006). Abnormal excitation of CH4: Meynert nucleus basalis may lead to abnormal permeability of the BBB, allowing SB to demonstrate the same muscarinic receptor blocking effects as scopolamine. No reports have indicated whether peripheral SB may enter the brain or demonstrate scopolamine‐like activity. However, in this clinical case, SB, similar to scopolamine, eliminated the flashbacks and nightmares (Jaffe et al., 2013), suggesting that the drug demonstrated a similar muscarinic receptor blockade.

Cholinergic nuclei, including the nucleus basalis of Meynert, are located at the confluence of the limbic and reticular activating systems, and they have widespread projections into the cortex and amygdala (Mesulam, 2013). Meynert's nucleus basalis receives dopaminergic input from the ventral tegmental area and substantia nigra, serotonergic input from the raphe nuclei, and noradrenergic input from the locus coeruleus. Its cholinergic contingent, Ch4, is the principal source of ACh in the cerebral cortex, hippocampus, and amygdala. In an experiment conducted on rats, Tonegawa et al. (2014) reported that memories of unpleasant events stored in the hippocampus can be eliminated by experiencing pleasant events; however, memories of unpleasant events that are stored in the amygdala cannot be erased. The amygdala plays a key role in the mechanisms leading to PTSD, and Meynert's nucleus basalis is closely connected to the amygdala in a bidirectional manner (Arita, 2006; Mesulam, 2013, Tonegawa et al., 2014). This makes it likely to be deeply involved in the generation of flashbacks and nightmares. In a study on the onset mechanism of intrusive memories (flashbacks) using fMRI, Bourne (2013) reported that flashbacks are associated with a widespread increase in activation, including in the amygdala, striatum, rostral anterior cingulate cortex, thalamus, and ventral occipital cortex. Liberzon (2008) reported that the medial prefrontal cortex, amygdala, sublenticular extended amygdala, and hippocampus mediate symptom formation in PTSD. Fitzgerald (2018) showed that emotional dysregulation in PTSD develops from complications within a large neurocircuitry involving the amygdala, insula, hippocampus, anterior cingulate cortex, and medial prefrontal cortex. However, the ACh‐based basal forebrain located at the rear end of the forebrain cortex was not examined using fMRI; thus, the relationship between PTSD and the ACh‐based basal forebrain was not addressed.

Trihexyphenidyl administration was highly effective in reducing both flashbacks and nightmares, and PTSD is thought to involve ACh. McLay (2007) showed that PTSD‐like symptoms are induced by an increase in ACh release caused by acetylcholinesterase inhibitors. A patient who had served during the Vietnam War and suffered from PTSD later suffered cerebral infarction and dementia. Upon administration of acetylcholinesterase inhibitors to improve his dementia, the patient exhibited PTSD‐like symptoms caused by an increase in ACh release. Anticholinergics have been suggested to help improve fear memory‐related symptoms in PTSD. In an experiment conducted on rats, Mitsushima et al. (2013) found that when fear memories are formed, α‐amino‐3‐hydroxy‐5‐methyl‐4‐isoxazolepropionic acid glutamate receptors are delivered to the synapses formed within the CA3 and CA1 regions of the hippocampus, and this delivery was mediated by increased ACh release. One potential mechanism of action of TP is the inhibition of the elevated release of ACh caused by the abnormal release of ACh in memory‐related structures, which is a hypothetical mechanism underlying flashback onset. In a recent study of TP in a rodent PTSD model, a single TP treatment prevented development of PTSD‐like behavior and inhibited aversive fear memory formation (Kaur et al., 2020). The results of the current trials suggest that TP can also reduce flashbacks after formation, likely through a similar anticholinergic effect on memory. As flashbacks improved, nightmares became more simultaneous and similar to flashbacks. This increased simultaneity and equivalence during recovery suggest that the mechanisms underlying both nightmares and flashbacks in the brain are potentially related to the effect of ACh on memory.

Trihexyphenidyl was also effective in relieving nightmares in patients with PTSD. Cholinergic activity is associated with REM sleep and the state of sleep during which dreams occur. In an experiment involving cats, Jasper (1971) found that the rate of release of free Ach during slow‐wave sleep (1.2 nanograms per minute per square centimeter of cortical surface) increased during REM sleep (2.2 nanograms per minute) and arousal (2.1 nanograms per minute). They also reported that ACh release was greater during REM sleep than during arousal. This increase in ACh release is possibly due to the propagation of POG waves by Ch5 and Ch6 neurons in the pons (American Academy of Sleep Medicine, 2005). Kobayashi (2007) reported polysomnographically measured sleep abnormalities in PTSD, and patients with R‐PTSD had greater REM density than healthy individuals. Singareddy (2002) showed that polysomnographic investigations frequently report REM sleep abnormalities in patients with PTSD. Unlike the regular REM sleep of healthy individuals, there is likely an abnormally elevated release of ACh during REM sleep in patients with PTSD. Patients administered TP reported nonvisual dream experiences, in which the dreams were felt to occur without a sense of visual perception. We did not find any reports on this phenomenon. According to the activation‐input source‐neuromodulation hypotheses of Hobson (1977, 2000), ACh neurotransmission from CH5 and CH6 during REM sleep ultimately reaches the occipital lobe. We hypothesize that the anticholinergic effects of TP initially act on memory‐related structures, such as the hippocampus and amygdala, and later the occipital lobe, leading to nonvisual dreams. Of the 30 investigated individuals, 25 (83%) endorsed this phenomenon. Future imaging studies can further elucidate the mechanisms underlying these nonvisual dream experiences.

Trihexyphenidyl showed remarkable effects on R‐PTSD‐related flashbacks and nightmares, with greater efficacy and faster action than anticipated. The patients' quality of life improved; however, two cases had been stable, but their PTSD symptoms worsened due to exacerbation of external factors. One patient, who was sexually abused by the father in the past, was hospitalized for translocation therapy, but for personal reasons had to live in the father's house, which led to the deterioration of their PTSD. Similarly, another patient who had been violently abused by the mother in the past had nightmares and flashbacks on the day she called as well as on the following day. Hospitalized patients showed improvements after 3–4 days of translocation therapy. Therefore, psychotherapy is an important addition to TP drug therapy. Other patients who were part of the trial were also taking concomitant psychiatric medications, and a few patients were taking appropriate psychiatric drugs and energetically adapting to their daily and social lives. Based on these initial results, TP may be an important therapeutic add‐on for R‐PTSD flashbacks and nightmares.

The present study had some limitations. First, we were unable to conduct a randomized controlled trial because of the limited number of patients with refractory disease at the clinic. We conducted an open‐label trial (*n = *22) to gain initial experience with a novel mechanism of action and a single‐blind trial (*n = *12) to further expand on this experience. Patients enrolled in our trial randomly visited our clinic between 2012 and 2019 and were refractory to drug treatment. To explore the potential bias of these designs on the results, we conducted regression analyses that aggregated patients and explored the impact of study design and TP on the results. Interestingly, while the study design did not affect the treatment effect of TP on CAPS scores, there was a significant interaction between TP treatment and study design in IES‐R scores, although each study separately showed a significant effect of TP on IES‐R scores. These results suggest that the IES‐R, as a self‐reported instrument, may amplify treatment effects during open‐label studies. Future studies should focus on clinician‐administered scales, such as the CAPS, and include self‐reported scales as secondary endpoints.

TP was added without removing any other drugs because we believed that it may be difficult to determine the action of TP due to withdrawal effects. We also acknowledge the possibility that the initial effect in Patient A following treatment with SB may be a placebo effect or an effect of other medical treatments; SB has a low brain penetration, although we cannot rule out the possibility that SB has a central effect even with this limited brain penetration. Because the study was conducted at a single site, it is difficult to generalize these findings to a broader population or in other clinical settings. Despite these limitations, the rapid and potent effects of TP in the subsequent open‐label trial and SBT emphasize the need for larger placebo‐controlled randomized controlled trials to validate the effects we observed.

## 4 CONCLUSIONS

- A case report showed that R‐PTSD‐related flashbacks resolved 20 min after receiving SB, a peripheral anticholinergic agent.
- TP, a centrally acting anticholinergic drug, has efficacy and a rapid onset in the treatment of R‐PTSD‐related nightmares and flashbacks
- To the best of our knowledge, this is the first pharmacological report describing the novel use of TP for R‐PTSD‐related nightmares.
- TP is an existing drug that has been used for over 65 years and is inexpensive and has no adverse effects.
- We strongly support the potential of repurposing TP for the treatment of PTSD‐related nightmares and flashbacks.

## 5 SUBJECTS AND METHODS

### 5.1 Trial institution

The trial institution was the Sogo PTSD Institute of the Sogo Clinic in Hiroshima City, Japan. The study administrators were Katsumasa Sogo (MD), Masanobu Sogo (MD), Yoshie Okawa (Pharm D), two nurses, and two medical staff. Each study administrator evaluated the physical and mental status and general vital signs of all patients. In the event of an emergency, the patients were instructed to call their respective clinician or physician. The trial results have been safely and privately kept at the Sago clinic, where only authorized personnel are allowed access.

### 5.2 Ethics

The present study was approved by the Ethics Committee of Warakukai Corporation on 30 November 2012 (Gen Ooi, MD, Chairman of the Ethics Committee, Professor Emeritus of the University of Tokyo, and the former Director of the National Institute for Environmental Studies; Clinical Trial Registry: UMIN trial ID, UMIN000028461). Patients with R‐PTSD treated at our clinic between September 2012 and October 2019 were recruited for the trial, which was completed in October 2019. They were provided informed consent forms, as per the WHO guidelines, to be filled before the trial.

### 5.3 Study subjects

The study included 34 patients examined at our clinic between 2012 and 2019 who had been diagnosed with R‐PTSD based on the DSM‐5. The included patients were aged 18–55 years (for anticholinergics) and had total bilirubin levels of less than 20 mg/dl and a platelet count of 70,000 mm or higher. The exclusion criteria were as follows: (a) patients treated with the same drug in the past; (b) patients who had already participated in other trials within the last 6 months; (c) patients with dementia; and (d) patients who were pregnant or lactating.

Twenty‐two patients were recruited in the open‐label trial, whereas the remaining 12 patients were enrolled in the SBT. Two patients experienced nightmares but not flashbacks; in contrast, two other patients experienced flashbacks but not nightmares. Other patients with PTSD experienced nightmares and flashbacks. Accordingly, most patients with PTSD and nightmares also experience flashbacks. At the initial consultation, the patients' ages ranged from 22 to 44 years. The time of onset ranged from 22 to 52 years, the mean duration from onset to treatment at our clinic was 5 ± 7 years, and the male‐to‐female ratio was 4:30.

Some studies use the term TR‐PTSD for R‐PTSD (Schoenfeld et al., 2013; Dunlop et al., 2014; Peuskens, 1999; Sackeim, 2001); however, the definition of “treatment resistance” is yet to be established. As mentioned before, patients with R‐PTSD received multiple long‐term treatments with several psychopharmaceuticals that were considered effective but were not found to be effective for nightmares and flashbacks. These patients continued with any medications they had been taking at enrollment. Most patients with PTSD had other concurrent mental disorders, including schizophrenia (*n = *3), Asperger's syndrome (*n = *2), and attention‐deficit and hyperactivity disorder (*n = *1).

### 5.4 Intervention

During the week following enrollment, the patients were administered TP at a dose of 2 mg two or three times a day (morning, noon, and night or morning and night), with night doses 1 hr before bedtime. The patients were asked to visit the hospital after 2 weeks of evaluation. Patients reported effects within 1–1.5 hr after TP administration; therefore, a prolonged evaluation period of several weeks was not required for TP. TP was administered for 1–3 weeks.

### 5.5 Evaluation

We analyzed the treatment outcomes of 34 patients who were administered TP, for whom the results were already known (open‐label trial). The 12 patients enrolled in the SBT were evaluated using the CAPS and Impact of Event Scale‐Revised (IES‐R). The general clinical features of the patients were determined by evaluating the overall effects using the Japanese version of the IER‐S (Asukai et al., 2002).

The gold standard assessment method for PTSD (1995) was used in this study. It is a 30‐item structured interview that can be used for current (past month or week) and lifetime diagnoses of PTSD. Of the questions, three pertained to memory. Item 1 (B1) is explained as recurrent, involuntary, intrusive, and distressing memories of traumatic events. Item 2 (B2) refers to recurrent distressing dreams with content and/or effects related to events. Item 3 (B3) includes dissociative reactions (e.g., flashbacks) characterized by individual feelings or simulating the recurrence of traumatic events (such reactions may occur on a continuum, with the most extreme expression being a complete loss of awareness of the present surroundings). B2 and B3 were used to evaluate the effect of TP on nightmares and flashbacks, respectively. The responses were evaluated according to frequency (0 = never, 1 = once or twice a month, 2 = once or twice a week, 3 = several times a week, and 4 = daily or almost daily) and intensity (0, none; 1, mild; 2, moderate; 3, severe; and 4, extreme).

Complete remission was characterized by the resolution of nightmares and flashbacks for at least 3 months, and the patients reported having good interpersonal relationships, had been successfully rehabilitated, and were constantly stable mentally and physically for at least 3 months. Furthermore, partial remission was characterized by a general improvement in the patients after drug administration, even though the nightmares and flashbacks had not resolved but had considerably improved. A CAPS score of 0–1 indicated complete remission, whereas a score of 2 indicated partial remission. All patients experienced significant stress that worsened when they recalled memories of stressful past events. Blood tests, electrocardiography, intraocular pressure measurements, and electroencephalography were regularly performed for all 34 patients who were administered TP.

### 5.6 Statistical analysis

Statistical analyses were performed for B2 (nightmares) and B3 (flashbacks) using CAPS and IES‐R. B2 and B3 are memory items in the CAPS test, and IES‐R is a psychological test aimed at creating a detailed picture of individual PTSD cases. We analyzed the pre‐ and postadministration outcomes using the Wilcoxon signed‐rank test and SPSS 24 software (IBM SPSS Statistics).

## CONFLICT OF INTEREST

The authors declare no conflicts of interest.

## AUTHOR CONTRIBUTIONS

Katsumasa Sogo, MD, Masanobu Sogo, MD, and Yoshie Okawa, Pharm D, are the authors of this study and were responsible for the literature search, study design, data collection, data analysis, data interpretation, and manuscript writing.

### PEER REVIEW

The peer review history for this article is available at https://publons.com/publon/10.1002/brb3.2147.

## Data Availability

The data that support the findings of this study are available upon request from the corresponding author. The data are not publicly available because of privacy or ethical restrictions.

## References

[brb32147-bib-0004] American Academy of Sleep Medicine (2005). International classification of sleep disorders (2nd ed.). Diagnostic and Coding Manual. American Academy of Sleep Medicine.

[brb32147-bib-0005] American Psychiatric Association (2013). Diagnostic and statistical manual of mental disorders (5th ed.). American Psychiatric Association.

[brb32147-bib-0006] Andreasen, N. C. , Carpenter, W. T. , Kane, J. M. , Lasser, R. A. , Marder, S. R. , & Weinberger, D. R. (2005). Remission in schizophrenia: Proposed criteria and rationale for consensus. American Journal of Psychiatry, 162(3), 441–449. 10.1176/appi.ajp.162.3.441 15741458

[brb32147-bib-0007] Arita, H. (2006). System neurophysiology of substances in the brain: Neurosciences of mentality and vigor. Corporation Chugai‐Igakusha.

[brb32147-bib-0008] Aserinsky, E. , & Kleitman, N. (2003). Regularly occurring periods of eye motility, and concomitant phenomena, during sleep. Journal of Neuropsychiatry Clinic Neuroscience, 15(4), 454–455. 10.1176/appi.neuropsych.15.4.454 14627774

[brb32147-bib-0009] Asukai, N. , Kato, H. , Kawamura, N. , Kim, Y. , Yamamota, K. , Kishimoto, J. , Miyake, Y. , & Nishizona‐Maher, A. (2002). Reliability and validity of the Japanese‐language version of the Impact of Event Scale‐Revised (IES‐R‐J): Four studies on different traumatic events. Journal of Nervous and Mental Disease, 190(3), 175–182. 10.1097/00005053-200203000-00006 11923652

[brb32147-bib-0010] Atwoli, L. , Stein, D. J. , Koenen, K. C. , & McLaughlin, K. A. (2015). Epidemiology of posttraumatic stress disorder: Prevalence, correlates and consequences. Current Opinions in Psychiatry, 28(4), 307–311. 10.1097/yco.0000000000000167 PMC4452282 26001922

[brb32147-bib-0016] Bourne, C. , Mackay, C. E. , & Holmes, E. A. (2013). The neural basis of flashback formation: The impact of viewing trauma. Psychological Medicine, 43(7), 1521–1532. 10.1017/S0033291712002358 23171530 PMC3806039

[brb32147-bib-0022] Dunlop, B. W. , Kaye, J. L. , Youngner, C. , & Rothbaum, B. (2014). Assessing treatment‐resistant posttraumatic stress disorder: The memory treatment resistant interview for PTSD (E‐TRIP). Behavioural Science (Basel), 4(4), 511–527. 10.3390/bs4040511 PMC4287702 25494488

[brb32147-bib-0024] Fitzgerald, J. M. , DiGangi, J. A. , & Phan, K. L. (2018). Functional neuroanatomy of emotion and its regulation in PTSD. Harvard Review of Psychiatry, 26(3), 116–128. 10.1097/hrp.0000000000000185 29734226 PMC5944863

[brb32147-bib-0025] Foa, E. B. , Keane, T. M. , Friedman, M. J. , & Cohen, J. A. (2009). Effective treatments for PTSD (2nd ed.). International Society for Traumatic Stress Studies.

[brb32147-bib-0026] Frank, E. , Prien, R. F. , Jarrett, R. B. , Keller, M. B. , Kupfer, D. J. , Lavori, P. W. , Rush, A. J. , & Weissman, M. M. (1991). Conceptualization and rationale for consensus definitions of terms in major depressive disorder: Remission, recovery, relapse, and recurrence. Archives of General Psychiatry, 48(9), 851–855. 10.1001/archpsyc.1991.01810330075011 1929776

[brb32147-bib-0029] Gross, J. J. (2008). Emotion regulation. In M. Lewis , J. M. Haviland‐Jones , & L. F. Barrett (Eds.), Handbook of Emotions (3rd ed., pp. 497–512). Guildford Press.

[brb32147-bib-0030] Hobson, J. A. , & McCarley, R. W. (1977). The brain as a dream state generator: An activation‐synthesis hypothesis of the dream process. American Journal of Psychiatry, 134(12), 1335–1348. 10.1176/ajp.134.12.1335 21570

[brb32147-bib-0031] Hobson, J. A. , Pace‐Schott, E. F. , & Stickgold, R. (2000). Dreaming and the brain: Toward a cognitive neuroscience of conscious states. Behavioral and Brain Sciences, 23(6), 793–842. 10.1017/S0140525X00003976 11515143

[brb32147-bib-0033] Ishikawa, R. , Uchida, C. , Kitaoka, S. , Furuyashiki, T. , & Kida, S. (2019). Improvement of PTSD‐like behavior by the forgetting effect of hippocampal neurogenesis enhancer memantine in a social defeat stress paradigm. Molecular Brain, 12(1), 68. 10.1186/s13041-019-0488-6 31370877 PMC6676601

[brb32147-bib-0034] Jaffe, R. J. , Novakovic, V. , & Peselow, E. D. (2013). Scopolamine as an antidepressant: A systematic review. Clinical Neuropharmacology, 36(1), 24–26. 10.1097/WNF.0b013e318278b703 23334071

[brb32147-bib-0035] Jasper, H. H. , & Tessier, J. (1971). Acetylcholine liberation from cerebral cortex during paradoxical (REM) sleep. Science, 172(3983), 601–602. 10.1126/science.172.3983.601 4324472

[brb32147-bib-0036] Kaur, H. , Kaur, R. , Jaggi, A. S. , & Bali, A. (2020). Beneficial role of central anticholinergic agent in preventing the development of symptoms in mouse model of post‐traumatic stress disorder. Journal of Basic and Clinical Physiology and Pharmacology, 31(6), 10.1515/jbcpp-2019-0196 32712590

[brb32147-bib-0039] Kobayashi, I. , Boarts, J. M. , & Delahanty, D. L. (2007). Polysomnographically measured sleep abnormalities in PTSD: A meta‐analytic review. Psychophysiology, 44(4), 660–669. 10.1111/j.1469-8986.2007.00537.x 17521374

[brb32147-bib-0042] Liberzon, I. , & Sripada, C. S. (2008). The functional neuroanatomy of PTSD: A critical review. Progress in Brain Research, 167, 151–169. 10.1016/s0079-6123(07)67011-3 18037013

[brb32147-bib-0043] McLay, R. N. , & Ho, J. U. (2007). Posttraumatic stress disorder‐like symptoms after treatment with acetylcholinesterase inhibitors. Journal of Neuropsychiatry and Clinical Neuroscience, 19(1), 92–93. 10.1176/jnp.2007.19.1.92 17308243

[brb32147-bib-0045] Mesulam, M. M. (2013). Cholinergic circuitry of the human nucleus basalis and its fate in Alzheimer's disease. Journal of Comparative Neurology, 52(18), 4124–4144. 10.1002/cne.23415 PMC4175400 23852922

[brb32147-bib-0046] Mitsushima, D. , Sano, A. , & Takahashi, T. (2013). A cholinergic trigger drives learning‐induced plasticity at hippocampal synapses. Nature Communications, 4, 2760. 10.1038/ncomms3760 PMC3831287 24217681

[brb32147-bib-0047] Morikawa, M. , Tanaka, Y. , Cho, H.‐S. , Yoshihara, M. , & Hirokawa, N. (2018). The molecular motor KIF21B mediates synaptic plasticity and fear extinction by terminating Rac1 activation. Cell Reports, 23(13), 3864–3877. 10.1016/j.celrep.2018.05.089 29949770

[brb32147-bib-0050] Nielsen, T. (2017). The stress acceleration hypothesis of nightmares. Frontiers in Neurology, 8, 201. 10.3389/fneur.2017.00201 28620339 PMC5451501

[brb32147-bib-0053] Pagel, J. F. (2000). Nightmares and disorders of dreaming. American Family Physician, 61(7), 2037–2042, 2044. 10779247

[brb32147-bib-0055] Peuskens, J. (1999). The evolving definition of treatment resistance. Journal of Clinical Psychiatry, 60(Suppl 12), 4–8. 10372602

[brb32147-bib-0056] Prien, R. F. , Carpenter, L. L. , & Kupfer, D. J. (1991). The definition and operational criteria for treatment outcome of major depressive disorder: A review of the current research literature. Archives of General Psychiatry, 48(9), 796–800. 10.1001/archpsyc.1991.01810330020003 1929769

[brb32147-bib-0057] Ramaswamy, S. , Madabushi, J. , Hunziker, J. , Bhatia, S. C. , & Petty, F. (2015). An open‐label trial of memantine for cognitive impairment in patients with posttraumatic stress disorder. Journal of Aging Research, 2015, 934162. 10.1155/2015/934162 26064685 PMC4443759

[brb32147-bib-0063] Sackeim, H. A. (2001). The definition and meaning of treatment‐resistant depression. Journal of Clinical Psychiatry, 62(Suppl 16), 10–17. 11480879

[brb32147-bib-0064] Sasaki, T. , Hashimoto, K. , Okawada, K. , Tone, J. , Machizawa, A. , Tano, A. , Nakazato, M. , & Iyo, M. (2013). Ifenprodil for the treatment of flashbacks in adolescent female posttraumatic stress disorder patients with a history of abuse. Psychotherapy and Psychosomatics, 82, 344345. 10.1159/000348585 23942427

[brb32147-bib-0068] Schoenfeld, F. B. , DeViva, J. C. , & Manber, R. (2012). Treatment of sleep disturbances in posttraumatic stress disorder: A review. Journal of Rehabilitative Research and Development, 49(5), 729–752. 10.1682/JRRD.2011.09.0164 23015583

[brb32147-bib-0072] Singareddy, R. K. , & Balon, R. (2002). Sleep in posttraumatic stress disorder. Annals Clinical Psychiatry, 14(3), 183–190. 10.3109/10401230209147455 12585568

[brb32147-bib-0077] Tonegawa, S. , Redondo, R. L. , Kim, J. , Arons, A. L. , Ramirez, S. , & Liu, X. (2014). Bidirectional reversal of the valence associated with a hippocampal contextual memory engram. Nature, 513(7518), 426–430. 10.1038/nature13725 25162525 PMC4169316

[brb32147-bib-0079] Van Horn, N. L. , & Street, M. (2019). Night Terrors. StatPearis Publishing. 29630274

[brb32147-bib-0081] Wahl, D. (1984). Pharmacokinetics of the substance in the rat. Documents from Boehringer Ingelheim Co., Ltd.

[brb32147-bib-0083] Weathers, F. W. , Blake, D. D. , Schnurr, P. P. , Kaloupek, D. G. , Marx, B. P. , & Keane, T. M. (2013). The Clinician‐Administered PTSD Scale for DSM‐5 (CAPS‐5). Retrieved from https://www.ptsd.va.gov 10.1037/pas0000486 PMC5805662 28493729

[brb32147-bib-0084] Wick, H. (1951). Buscopan. Naunyn Schmiedebergs Arch Exp Pathol Pharmakol, 213(6), 485–500. 14934141

